# Frame-Based CT Image Reconstruction via the Balanced Approach

**DOI:** 10.1155/2017/1417270

**Published:** 2017-09-17

**Authors:** Weifeng Zhou, Hua Xiang

**Affiliations:** ^1^College of Mathematics and Physics, Qingdao Science and Technology University, Qingdao, Shandong 266071, China; ^2^School of Computer Science and Technology, Shandong University, Jinan, Shandong 250101, China; ^3^College of Cooperation, Qingdao Agricultural University, Qingdao, Shandong 266109, China

## Abstract

Frame-based regularization method as one kind of sparsity representation method has been developed in recent years and has been proved to be an efficient method for CT image reconstruction. However, most of the developed CT image reconstruction methods are analysis-based frame methods. This paper proposes a novel frame-based balanced hybrid model with two sparse regularization terms for CT image reconstruction. We generalize the fast alternating direction method to solve the proposed model so that every subproblem can be easily solved. The numerical experiments suggest that the proposed hybrid balanced-based wavelet regularization scheme is efficient in terms of reducing the defined reconstruction root mean squared error and improving the signal to noise ratio in CT image reconstruction.

## 1. Introduction

X-ray computed tomography (CT) image reconstruction is an indispensable tool for diagnosing diseases and research requirements. However, X-ray radiation is harmful, and high-dose X-ray radiation may induce genetic mutation, cell canceration, and so on [1]. Therefore, more and more attentions are paid on the low-dose X-ray CT image reconstruction. Since X-ray CT imaging quality depends on the X-ray dose, reducing the X-ray dose will result to poor image reconstruction quality. Consequently, how to decrease the X-ray dose not to affect the diagnosis is a hot topic in recent years. Mathematically, CT image reconstruction often can be formulated as a linear inverse problem. For the detected measurements data *b*, the objective is to find the targeted image *u* from the following equation:

(1) $$\begin{array}{c} b=Au+\varepsilon , \end{array}$$

where *A* is the discrete radon linear transform operator and *ε* denotes the noise with variance *δ*.

One strategy for X-ray radiation-dose reduction is to reduce the projection data, but this few-view method will result in insufficient data. As a result of the undersampling of this strategy and the system errors, the above-mentioned problem (1) is usually ill-posed from the mathematical point of view. Therefore, traditional filtered back projection (FBP) method [2] cannot yield desirable imaging quality. Sparse regularization methods are developed in recent years to overcome the ill-posedness of these problems, and moreover, these methods can acquire higher quality images in few-view circumstance. The sparse representation methods assume that images are sparse in some transformed domains. Discrete gradient [3], that is, the so-called total variation (TV), is such a sparse transform domain to solve the few-view image reconstruction problems. Although the TV-based regularization sparse domain is useful in reducing radiation dose [4, 5], there are also some shortcomings, for example, the staircase effect [6], and the power of it is still limited [7]. Therefore, many improved methods are proposed, such as PWLS-TGV proposed by Niu et al. [8], TVS-POCS proposed by Liu et al. [9], and the method proposed by Ritschl et al. [10]. Besides, some other sparse transform domains are developed, such as the gamma regularization-based method [11], some nonlocal domains [12, 13], different kinds of wavelet frame domains [14–16], and some dictionary learning sparse methods [17, 18].

This paper mainly considers the sparse representation by wavelet tight frame. Wavelet tight frame can ensure the given signal be perfectly represented as a linear combination of the sparse wavelet coefficients which is also called the perfect reconstruction property [19]. Due to the flexibility of decomposition and reconstruction and the well performance, wavelet tight frame-based methods have been widely used in almost every branch of image processing [14, 20–22]. In recent years, most of the developed wavelet tight frame methods for CT image reconstruction are analysis-based wavelet frame methods. This paper proposes a novel balanced-based method. The remainder of this paper is organized as follows. In Section 2, we present some necessary preliminaries and theories about the wavelet tight frame.  Section 3 presents the proposed balanced model for CT image reconstruction and develops the efficient computational algorithm for solving the proposed strategy. Numerical simulations to demonstrate the improvement of our proposed method in terms of RMSE and PSNR are given in Section 4. In the end, we make the conclusion in Section 5.

## 2. Preliminaries

For convenience, we first present some basic definitions and some results of the wavelet tight frame used in the proposed model. More details can be seen in [20, 21].

In the 1D discrete circumstance, a set of vectors {*x*_*i*_ ∈ *ℝ* ^*M*^}_*i*=1_^*N*^(*N* ≥ *M*) is called a wavelet tight frame if for each *b* ∈ *ℝ* ^*M*^,

(2) $$\begin{array}{c} \overset{N}{\underset{i=1}{\sum }}{\left|\langle x_{i},b\rangle \right|}^{2}={\left\|b\right\|}_{2}^{2}, \end{array}$$

where 〈·, ·〉 denotes the inner product. The corresponding analysis operator denoted as *W* is written as

(3) $$\begin{array}{c} W={\left[x_{1},x_{2},\ldots ,x_{N}\right]}^{T}. \end{array}$$

Then, *Wb* = {〈*b*, *x*_*i*_〉}_*i*=1_^*N*^ is called the wavelet coefficients. Another operator *W* ^*T*^ which is usually called the synthesis operator is the synthesis of the wavelet coefficients, that is, if *c* denotes the wavelet coefficients, then

(4) $$\begin{array}{c} W^{T}c=\overset{N}{\underset{i=1}{\sum }}c\left(i\right)x_{i}. \end{array}$$

Then

(5) $$\begin{array}{c} W^{T}W=I_{M}, \end{array}$$

which can be derived by the identity (2). Here, *I*_*M*_ : *ℝ* ^*M*^ → *ℝ* ^*M*^ is the identity operator. This property is often called the “perfect reconstruction property,” which can reduce the calculation amount in some applications.

Based on the Unitary Extension Principle (UEP) condition [20], the wavelet tight frame often can be generated by some filters {*a*_*i*_}_*i*=0_^*r*−1^ that satisfy

(6) $$\begin{array}{c} \overset{r-1}{\underset{i=0}{\sum }}\sum_{n\in \mathbb{Z}}a_{i}\left(l+n\right)a_{i}\left(n\right)=\delta_{l},\;\forall l\in \mathbb{Z}. \end{array}$$

Here, *δ*_*l*_ = 1 when *l* = 0; otherwise it is zero. Piecewise linear B-spline framelet is such a wavelet tight frame whose associated filters are

(7) $$\begin{array}{c} a_{0}=\frac{1}{4}\left[1,2,1\right],\\ a_{1}=\frac{\sqrt{2}}{4}\left[1,0,-1\right],\\ a_{2}=\frac{1}{4}\left[-1,2,-1\right]. \end{array}$$

The wavelet-based method has been used in almost every image processing branch [22–24]. Two-dimensional wavelet tight frame filters can be obtained by the tensor product of the corresponding one-dimensional filters.

Given the observed data *b*; the sparse regularization methods for image processing based on the wavelet tight frame can be summarized as

(8) $$\begin{array}{c} x^{*}=\arg\underset{x}{\text{min}}\frac{1}{2}{\left\|{AW}^{T}x-b\right\|}_{2}^{2}+\frac{\kappa }{2}{\left\|\left(I-{WW}^{T}\right)x\right\|}_{2}^{2}+\lambda {\left\|x\right\|}_{p}, \end{array}$$

where *A* denotes a linear transform operator, which is a discrete radon transform in CT image reconstruction, a Fourier transform in MR image reconstruction, and a convolution operator in image deblurring. ‖·‖_*p*_ denotes the *p*-norm so as to finally obtain a sparse solution. ‖·‖_1_ as the approximation of ‖·‖_0_ is often used to realize the sparse regularization. Compared with ‖*x*‖_0_, ‖*x*‖_1_ is convex so that the well-posed property can be guaranteed. Then, the target image *u* ^∗^ = *W* ^*T*^ *x* ^∗^. Here, the middle term in (8), that is, (*κ*/2)‖(*I* − *WW* ^*T*^)*x*‖_2_^2^, is used to balance the distance between the target image *W* ^*T*^ *x* and the coefficient *x*. Then, in terms of different values of *κ*, three approaches are distinguished, that is, the analysis approach (*κ* = ∞), the balanced approach (0 < *κ* < ∞), and the synthesis approach (*κ* = 0). Obviously, the three approaches are the same when *W* is orthogonal. Generally speaking, it is difficult to make a conclusion that which approach among the three approaches described in (8) is better. Every approach has its own favorite image sets [25].

For the balanced-based approach, we have the following result [19].

**Lemma 1 .**

*Let W and W* ^*T*^ *, respectively, denote the analysis operator and the synthesis operator of a wavelet tight frame; then *‖(*I* − *WW* ^*T*^)*x*‖_2_^2^ + ‖*W* ^*T*^ *x* − *b*‖_2_^2^ = ‖*x* − *Wb*‖_2_^2^ *holds.*

## 3. The Proposed Model and Method

In recent years, most of the developed methods for CT image reconstruction are analysis-based frame methods [16, 21, 26], and many state-of-the-art methods such as Split Bregman method [27], alternative direction [28], and augmented Lagrangian method [29] are implemented to solve these problems. The researches of image reconstruction modeled based on balanced method [25] and synthesis method [30] are relatively few. Since every approach has its own favorite image sets, it is difficult to make a conclusion which one is better [25].

This paper proposes a novel-constrained balanced-based model CT image reconstruction as follows:

(9) $$\begin{array}{c} \underset{x}{\min}\frac{\kappa }{2}{\left\|\left(I-{WW}^{T}\right)x\right\|}_{2}^{2}+\frac{\alpha }{2}{\left\|W^{T}x\right\|}_{2}^{2}+\beta {\left\|x\right\|}_{1},\\ s.t.\;{\left\|{AW}^{T}x-b\right\|}_{2}^{2}\leq \delta^{2}, \end{array}$$

where *A* denotes a radon transform operator, *b* is the obtained data from the scanner, and *W* is the wavelet tight frame. Then, the reconstructed image *u* ^∗^ = *W* ^*T*^ *x* ^∗^, where *x* ^∗^ denotes the solution of (9). Compared with the model in (8), the added term (*α*/2)‖*W* ^*T*^ *x*‖_2_^2^ is used to regularize the solution further and avoid the Gibbs defects bring from the wavelet tight framelet. In general, the parameter *α* is chosen to be smaller than the parameter *β*. Actually, bigger *α* will result in the overall smoothness of the results' image. In recent years, some efficient algorithms are developed for the balanced-based models, for example, proximal linearized alternating direction method in which the linearization of quadratic term of the augmented function for the model with one regularization term was used [31].

Next, let us investigate the corresponding flexible iteration algorithm for our proposed balanced-based model. We also can use the fast alternating direction method for solving our proposed balanced-based model. The corresponding convergence analysis of the alternating direction method can resort to the [32, 33]. By introducing *W* ^*T*^ *x* = *u*, the constrained minimization problem (9) can be changed into the following unconstrained one:

(10) $$\begin{array}{c} \underset{u,x,z,f}{\min}\frac{\iota }{2}{\left\|Au-b+f\right\|}_{2}^{2}+\frac{\kappa }{2}{\left\|\left(I-{WW}^{T}\right)x\right\|}_{2}^{2}+\frac{\delta }{2}{\left\|u-W^{T}x+z\right\|}_{2}^{2}+\frac{\alpha }{2}{\left\|u\right\|}_{2}^{2}+\lambda {\left\|x\right\|}_{1}. \end{array}$$

Actually, (10) is equivalent to the following:

(11) $$\begin{array}{c} \underset{u,x,z,f}{\min}\frac{1}{2}{\left\|Au-b+f\right\|}_{2}^{2}+\frac{\tau }{2}{\left\|\left(I-{WW}^{T}\right)x\right\|}_{2}^{2}+\frac{\gamma }{2}{\left\|u-W^{T}x+z\right\|}_{2}^{2}+\frac{\mu }{2}{\left\|u\right\|}_{2}^{2}+v{\left\|x\right\|}_{1}, \end{array}$$

where *τ*, *γ*, and *μ* are parameters. Here, we omit the relationship between these parameters and the parameters in (10).

Here, we choose *τ* = *γ* for the convenience of calculation. Then, based on Lemma 1, (11) is equivalent to the following:

(12) $$\begin{array}{c} \underset{u,x,z,f}{\text{min}}\frac{1}{2}{\left\|Au-b+f\right\|}_{2}^{2}+\frac{\gamma }{2}{\left\|x-W\left(u+z\right)\right\|}_{2}^{2}+\frac{\mu }{2}{\left\|u\right\|}_{2}^{2}+v{\left\|x\right\|}_{1}. \end{array}$$

Then by the alternative direction method, the minimization problem (12) can be decomposed into the following four subproblems:

(13) $$\begin{array}{c} u^{k+1}=\arg\underset{u}{\min}\frac{1}{2}{\left\|Au-b+f^{k}\right\|}_{2}^{2}+\frac{\gamma }{2}{\left\|u-W^{T}x^{k}+z^{k}\right\|}_{2}^{2}+\frac{\mu }{2}{\left\|u\right\|}_{2}^{2}, \end{array}$$

(14) $$\begin{array}{c} f^{k+1}={Au}^{k+1}-b+f^{k}, \end{array}$$

(15) $$\begin{array}{c} x^{k+1}=\arg\underset{x}{\min}\frac{\gamma }{2}{\left\|u^{k+1}-W^{T}x+z^{k}\right\|}_{2}^{2}+v{\left\|x\right\|}_{1}, \end{array}$$

(16) $$\begin{array}{c} z^{k+1}=z^{k}+u^{k+1}-W^{T}x^{k+1}. \end{array}$$

As for the first subproblem, by the KKT condition, *u* ^*k*+1^ = (*A* ^*T*^ *A* + *γ* + *μ*)^−1^[*A* ^*T*^ *b* + *γ*(*W* ^*T*^ *x* ^*k*^ − *b*)] can be easily solved by the well-known conjugate gradient (CG) method. On one hand, the added term (*μ*/2)‖*u*‖_2_^2^ in (12) can regularize the solution further; on the other hand, we can see from the solution of the *u*-subproblem (13) that parameter *μ* can further overcome the ill-conditioning of the operator *A* in CT scanning. Then, we summarize our proposed algorithm based on balanced wavelet tight frame approach for CT image reconstruction as in Algorithm 1.

## 4. Numerical Simulations

In this section, two numerical studies are presented to illustrate the well performance of our proposed scheme. We compare our proposed hybrid regularization scheme (*μ* ≠ 0) in (12) with the traditional FBP algorithm and the one with only one regularization term (*μ* = 0) by our proposed balanced-based frame algorithm. In the following two simulations, we adapt the piecewise linear B-spline tight frame as the transform operator. For evaluating the quality of the reconstructed images, the following root mean squared error (RMSE) and the peak value signal to noise ratio (PSNR) are used:

(17) $$\begin{array}{c} RMSE=\frac{{\left\|u-f\right\|}_{2}}{{\left\|f\right\|}_{2}},\\ PSNR=20*{\text{log}}_{10}\frac{255}{\left(1/MN\right)*{\left\|u-f\right\|}_{2}}. \end{array}$$

Here, *u* and *f* denote the reconstructed image and the original image, respectively, and *M* and *N* denote the size of the image. Generally speaking, smaller RMSE means better reconstructed quality and higher PSNR means more closer to the original images. Some other appraisal criteria can also be used, such as the SSIM [34]. The iteration is stopped when ‖*u* ^*k*+1^ − *u* ^*k*^‖_2_/‖*u* ^*k*^‖_2_ ≤ 10^−3^. In these experiments, we use GPU (graphics processing units) to accelerate the computation of the *A* and *A* ^*T*^ for more fast reconstruction [35]. In the following simulations, the projection views were equally distributed over 360° and detector offset was not to be considered. All the experiments are performed by Matlab 2009 on the PC with 64-bit operating system and 2.90 GHz processor. In addition, after a great deal of our simulations, we found that the parameter *μ* should be much smaller than the parameter *v*, since larger *μ* will result in the overall smoothness of the reconstruction image. The parameter *γ* can be set as 5 or 6 when the noise and the projection views are not changed greatly. The stronger the noise, the greater the parameter *v* should be used. Next, we will present two concrete examples.

**Example 4.1.**

**Figure 1:**
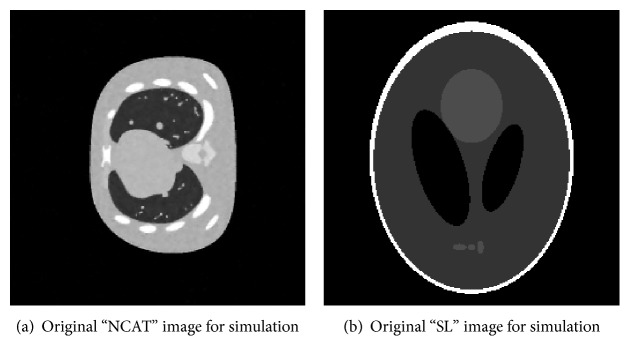
Original images used for simulation.

**Figure 2:**
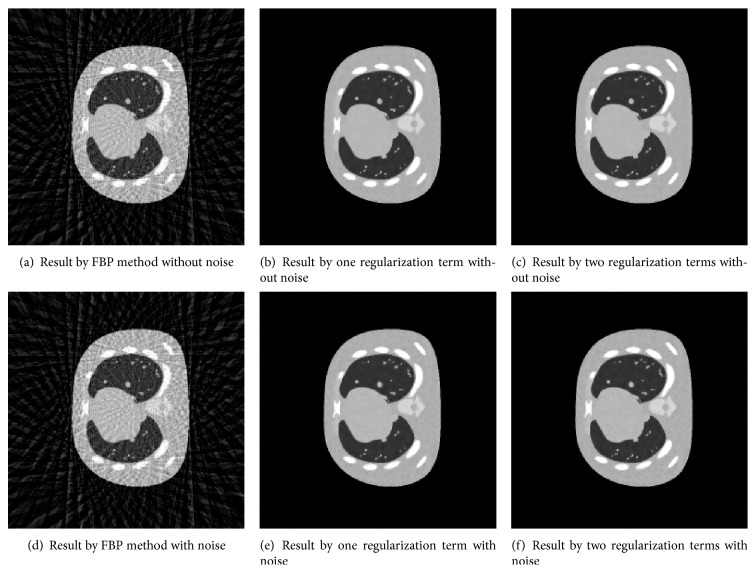
Recovered results by FBP method and models with two regularization terms and one regularization term based on our proposed balanced approach with 50 projection views.

We use “NCAT” shown in Figure 1(a) to evaluate the proposed model and algorithm. In recent years, “NCAT” has been widely used to evaluate medical imaging technology. The reconstructions RMSE and PSNR by FBP method (Figure 2(a)) with 50 projection views are 0.2701 and 69.0921, respectively. Figures 2(b) and 2(c) are, respectively, the recovered results with 50 projection views by models with one regularization (*μ* = 0) and two regularizations (*μ* ≠ 0) in (12) based on our proposed balanced approach with algorithm parameters *γ* = 6, *v* = 0.2, and *μ* = 0.01. These parameters are optimized. 23 iterations are carried out with about 3.6 s. The values of RMSE and PSNR are 0.0271 and 89.0138, respectively, for two regularization term balanced algorithm and 0.0305 and 87.9635, respectively, for the corresponding one regularization term balanced algorithm. We can see that in terms of RMSE, 12.5% error was reduced with the added regularization terms (*μ*/2)‖*u*‖_2_^2^. The reconstruction quality is more pleasing for the balanced algorithm with two regularization term than the corresponding balanced algorithm with one regularization term.

Due to the device defect or the low-exposing dose, noise is often inevitable. So, we add 1% noise to the projection data of “NCAT.” The corresponding reconstructions RMSE and PSNR by FBP method (Figure 2(d)) are 0.2751 and 68.8931, respectively. The corresponding reconstructions RMSE and PSNR are 0.0338 and 87.1018, respectively, for two regularization term balanced-based algorithm (Figure 2(f)) and 0.0362 and 86.2801, respectively, for the one regularization term balanced-based algorithm (Figure 2(e)). Consequently, in the environments both with noise and without noise, the balanced algorithm with two regularization terms can obtain more better reconstruction quality in terms of RMSE and PSNR.

**Example 4.2.**

**Figure 3:**
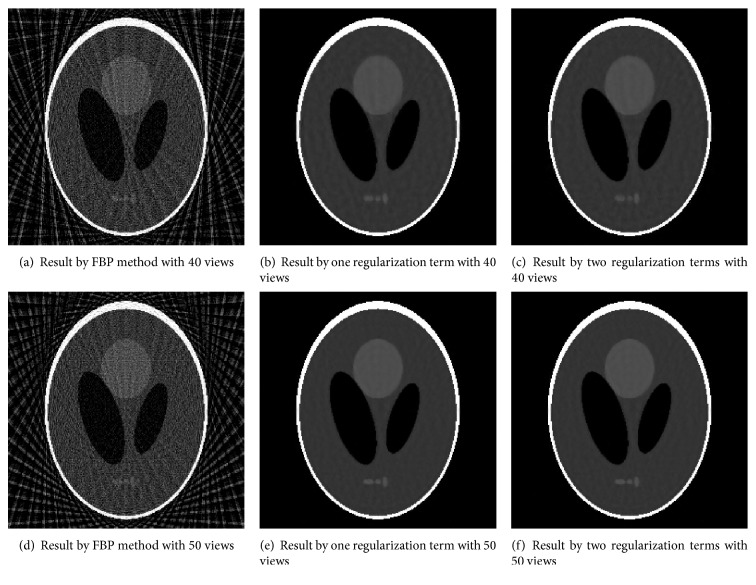
Recovered results by FBP method and models with two regularization terms and one regularization term based on our proposed balanced approach under different projection views.

Shepp-Logan image “SL” as our second test image can be seen in Figure 1(b). We evaluate algorithms under different views. The corresponding reconstructions RMSE and PSNR by FBP method with 40 projection views (Figure 3(a)) are 0.5906 and 64.8555, respectively. The corresponding reconstructions RMSE and PSNR by FBP method with 50 projection views (Figure 3(d)) are 0.5726 and 65.1283, respectively. Obviously, the streak artifacts are introduced by FBP algorithm with few-view projections. Figures 3(c) and 3(d) are, respectively, the recovered results with 40 projection views by models with one regularization (*μ* = 0) in (12) and two regularizations (*μ* ≠ 0) in (12) based on our proposed balanced approach with algorithm parameters *γ* = 5, *v* = 0.2, and *μ* = 0.01. Every parameter has been optimized. The values of RMSE are 0.0780 and 0.0759. The values of PSNR are 82.1841 and 82.6393. The reconstruction results with 50 projection views are displayed in Figures 3(e) and 3(f). The values of RMSE are 0.0581 and 0.0557. The values of PSNR are 84.2616 and 85.9924. The RMSE and PSNR of our proposed hybrid regularization method are improved under different views which means more pleasing reconstruction results. Both the two kinds of the balanced-based image reconstruction schemes yield more desiring results than the FBP method.

## 5. Conclusion

In this paper, we propose a balanced-based wavelet CT reconstruction model with two regularization term. We investigate the fast algorithm for the proposed hybrid model based on the alternative direction method. Simulation results evidently demonstrate the superiority of our proposed scheme in reducing the values of RMSE and promoting the PSNR. This method is very flexible and can also be easily generalized to some other image processing problems.

## Figures and Tables

**Algorithm 1:**
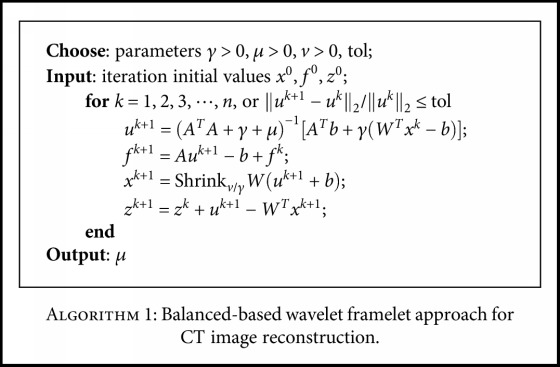
Balanced-based wavelet framelet approach for CT image reconstruction.
